# A Review of Photocatalysts Prepared by Sol-Gel Method for VOCs Removal

**DOI:** 10.3390/ijms11062336

**Published:** 2010-05-28

**Authors:** Ting Ke Tseng, Yi Shing Lin, Yi Ju Chen, Hsin Chu

**Affiliations:** Department of Environmental Engineering and Sustainable Environment Research Center, National Cheng Kung University, 1 University Road, Tainan 701, Taiwan

**Keywords:** sol-gel, photocatalytic oxidation, VOCs

## Abstract

The sol-gel process is a wet-chemical technique (chemical solution deposition), which has been widely used in the fields of materials science, ceramic engineering, and especially in the preparation of photocatalysts. Volatile organic compounds (VOCs) are prevalent components of indoor air pollution. Among the approaches to remove VOCs from indoor air, photocatalytic oxidation (PCO) is regarded as a promising method. This paper is a review of the status of research on the sol-gel method for photocatalyst preparation and for the PCO purification of VOCs. The review and discussion will focus on the preparation and coating of various photocatalysts, operational parameters, and will provide an overview of general PCO models described in the literature.

## 1. Introduction

Since the research of photocatalytic water split on TiO_2_ electrodes was conducted in 1972 [1], the photocatalytic oxidation of aqueous and gaseous contaminants has been extensively studied. It has drawn considerable academic interest as a very attractive, nonselective, room-temperature process for the degradation of organic pollutants [2–4]. It is a process where the illumination of a semiconductor forms photoexcited electrons and holes (in a vacant conduction band) that will react with contaminants adsorbed on the photocatalyst surface. Semiconductors, mainly by virtue of their electronic configuration, can provide light-induced charges for redox processes. In particular, they are characterized by a filled valence band and an empty conduction band. The elementary mechanisms of photocatalytic transformation include a number of steps, which have been exhaustively described [2–4]. In contrast with other semiconductors (*i.e.*, WO_3_, ZnO, ZnS, Fe_2_O_3_, CdS, and SrTiO_3_), TiO_2_ is widely used in environmental applications because of its physical and chemical stability, lower cost, non-toxicity and resistance to corrosion. For the practice of photocatalytic reactions, it is necessary to have light at a sufficient intensity so as to possess energy that exceeds the TiO_2_ band gap energy (*E*_bg_). For the two crystal structures of TiO_2_, *E*_bg_ (anatase) = 3.2 eV, and *E*_bg_ (rutile) = 3.02 eV, the absorption thresholds correspond to 380 and 410 nm, respectively [5]. However, the absorption wavelength of anatase does not conform to the solar spectrum region; the solar energy of about 3.0 eV (λ ≤ 410 nm) is less than 5% [6]. Consequently, the light-used efficiency of traditional photocatalysts is less than 5%, which results in low photoreaction rates and therefore limits the commercial potential. A number of studies aim at overcoming this problem by focusing on the surface modifications of photocatalysts. The strategies of modification are: (i) inhibiting recombination by increasing the charge separation and therefore the efficiency of the photocatalytic process; (ii) increasing the wavelength response range (*i.e.*, the photocatalyst can be excited in the visible light region); and (iii) changing the selectivity or yield of a particular product [4]. In recent years, the technique of metal ion-doped into TiO_2_ has been widely studied. Noble metals, e.g., Pt, are most commonly investigated [4,7], and other metals, e.g., Au, Pd, Ru, have been reported to be beneficial for photocatalytic reactions [7]. The transitional metal ions, e.g., Fe [3], have been used for increasing the photocatalytic activity. The sol-gel technique has been reviewed by a number of investigators [8,11]. The advantages of the sol-gel method are: (i) it is easy to operate and is inexpensive; (ii) the films are readily anchored on the substrate; and (iii) it can be used for the deposition of substrates that have complex surfaces or large surface areas.

The air purification technique of photocatalytic oxidation (PCO) commonly uses nanosemiconductor catalysts and ultraviolet (UV) light to convert organic compounds in indoor air into benign and odorless constituents – water vapor (H_2_O) and carbon dioxide (CO_2_) [12]. Most of the PCO reactors use nano-titania (TiO_2_) as the catalyst that can be activated by UV light. A schematic of the UV PCO process of VOCs using TiO_2_ as the catalyst was shown in the previous literature [13]. An electron in an electron-filled valence band (VB) is excited by photoirradiation to a vacant conduction band (CB), leaving a positive hole in the VB. These electrons and positive holes will drive the reduction and oxidation, respectively, of compounds adsorbed on the surface of a photocatalyst [14].

The activation equation can be written as:

(1) $${\text{TiO}}_{2}+\text{h}\nu \to {\text{h}}^{+}+{\text{e}}^{-}$$

In this reaction, h^+^ and e^−^ are powerful oxidizing and reducing agents, respectively.

The oxidation and reduction reactions can be expressed as:

(2) $$\text{Oxidation}\;\text{reaction}:\;{\text{h}}^{+}+{\text{OH}}^{-}\to \text{OH}•$$

(3) $$\text{Reduction}\;\text{reaction}:\;{\text{e}}^{-}+{\text{O}}_{2\;\text{ads}}\to {\text{O}}_{2\;\text{ads}}^{-}$$

When organic compounds are chemically transformed by a PCO device, it is the hydroxyl radical (OH•), derived from the oxidation of adsorbed water or adsorbed OH^−^, that is the dominant oxidant. Its net reaction with a VOC can be expressed as:

(4) $$\text{OH}•+\text{VOC}+{\text{O}}_{2}\to {\text{nCO}}_{2}+{\text{mH}}_{2}\text{O}$$

The process of PCO has several advantages [15]: (i) it is GRAS (Generally Recognized As Safe): the common photocatalyst is anatase TiO_2_, an n-type semiconductor oxide which is also a component of some toothpastes and pharmaceutical suspensions; (ii) it is a mild oxidant: kinetic studies demonstrate that the ultimate source of oxygen during oxidation is molecular oxygen, a far milder oxidant than hydrogen peroxide or ozone, *etc*.; (iii) it can be used in ambient temperature: photocatalysis appears to be active at room temperature; (iv) it possesses generality: while several mechanistic pathways for oxidation have been proposed, the dominant view is that the hydroxyl radical (or some other equally strong oxidant) is photogenerated on the titania surface. The potency of this oxidant is responsible for the titania’s broad activity toward various contaminants (such as aromatics, alkanes, olefins, halogenated hydrocarbons, odor compounds, *etc*.).

## 2. Sol-Gel Methods

The sol-gel process is a wet-chemical technique widely employed recently in the fields of materials science and ceramic engineering. Such methods are utilized primarily for the fabrication of materials (typically a metal oxide) starting from a chemical solution which acts as the precursor for an integrated network (or gel) of either discrete particles or network polymers.

Typical precursors are metal alkoxides and metal chlorides, which undergo various forms of hydrolysis and polycondensation reactions. The formation of a metal oxide involves connecting the metal centers with either oxo (M–O–M) or hydroxo (M–OH–M) bridges, and generating metal-oxo or metal-hydroxo polymers in solution. Thus, the sol evolves towards the formation of a gel-like diphasic system containing both liquid and solid phases whose morphologies range from discrete particles to continuous polymer networks.

In the case of the colloid, the volume fraction of particles (or particle density) may be so low that a significant amount of fluid may need to be removed initially for the gel-like properties to be recognized. This can be accomplished through plenty of ways. The simplest method is to allow time for sedimentation to occur, and then pour off the remaining liquid. Centrifugation can also be used to accelerate the process of phase separation.

Removal of the remaining liquid (solvent) phase requires a drying process, which is typically accompanied by a significant amount of shrinkage and densification. The rate at which the solvent can be removed is ultimately determined by the distribution of porosity in the gel. The ultimate microstructure of the final component will be strongly influenced by changes imposed upon the structural template during this phase of processing. Afterwards, a thermal treatment, or a firing process, is also necessary in order to favor further poly-condensation and to enhance the mechanical properties and structural stability via final sintering, densification, and grain growth. One of the distinct advantages of this methodology, as opposed to the more traditional processing techniques, is that densification is often achieved at much lower temperatures.

The precursor sol can be either deposited on a substrate to form a film (e.g., by dip coating or spin coating), casted into a suitable container with the desired shape (e.g., to obtain monolithic ceramics, glasses, fibers, membranes, and aerogels), or used to synthesize powders (e.g., microspheres, nanospheres). The sol-gel approach is a cheap and low-temperature technique finely controls the product’s chemical composition. Even small quantities of dopants, such as organic dyes and rare earth elements, can be introduced in the sol and end up uniformly dispersed in the final product. It can be used in ceramics processing and manufacturing as an investment casting material, or as a means of producing very thin films of metal oxides for various purposes. Sol-gel derived materials have diverse applications in optics, electronics, energy, space, bio-sensors, medicine (e.g., controlled drug release), and in reactive material and separation (e.g., chromatography) technology [16,17].

The sol-gel approach provides a resourceful way of synthesizing inorganic polymer and organic-inorganic hybrid materials. Historically, the use of sol-gel technology has been introduced in the mid-1800s [18]. This technology was used almost one century later by the Schott Glass Company (Jena, Germany) [18]. Sol-gel process can be applied under extraordinarily mild conditions; thus, it can be used to obtain products of various sizes, shapes and formats (e.g., fibers, films, monoliths, and nano-sized particles). Sol-gel technology has found increasing applications in the development of new materials for catalysis [19,20], chemical sensors [21,22], membranes [23], fibers [24], optical gain media [25], photochromic and non linear applications [26–28], and in solid state electrochemical devices [29]. The technology is utilized in a diverse range of scientific and engineering fields, such as the ceramic industry [18], nuclear field industry [18], and electronics industry [30]. The inherent advantages of the sol-gel process are summarized as follows [31]:

1. Better homogeneity from raw materials.
2. Better purity from raw materials.
3. Lower temperature of preparation.
4. Good mixing for multi-component systems.
5. Effective control of particle size, shape, and properties.
6. Better products from the special properties of the gel.
7. The creation of special products such as films.
8. The creation of new non-crystalline solids outside the range of normal glass formation.
9. The fine tuning of chromatographic selectivity via the possibility of creating hybrid organic-inorganic materials.
10. The possibility of designing the material structure and property through the proper selection of sol-gel precursor and other building blocks.
11. The possibility of achieving enhanced stationary phase stability and performance in chromatographic separations.

The sol-gel process is acid-catalyzed, and the sol-gel approach to column technology provides an effective means of chemically binding chromatographic stationary phases to the column inner surface. The sol-gel approach brought new promise of providing high stationary phase stability and column efficiency in separations. These early works stimulated further developments in the area of sol-gel stationary phases in HPLC [32–34], GC [35,36] and electrophoretic separations [30,37].

## 3. Fundamental Chemical Reactions in the Sol-Gel Process

Metal alkoxides are members of the family of organometallic compounds, which have one or more metal atoms in the molecule of the organic compounds. Metal alkoxides (R–O–M) like alcohols (R–OH), have a metal atom, M, replacing the hydrogen H in the hydroxyl group. They constitute the class of chemical precursors most widely used in sol-gel synthesis.

The most common mineral in the earth’s crust is silicon dioxide (or silica), SiO_2_. There are at least seven different crystalline forms of silica, including quartz. The basic building block of all of these crystalline forms of silica is the SiO_4_ tetrahedron. Since each tetrahedron shares 2 of its edges with other SiO_4_ tetrahedrals, the overall ratio of oxygen to silicon is 2:1 instead of 4:1. The intricate and highly specific geometry of this network of tetrahedrals takes years to form under incredible terrestrial pressures at great depth. That is why SiO_2_ is such a good building block for glass. Crystallization in a reasonable amount of time under the most ideal laboratory conditions is highly unlikely. Thus, amorphous silica is the major component of window glass.

A well studied alkoxide is silicon tetraethoxide, also known as tetraethyl orthosilicate (TEOS). The chemical formula for TEOS is given by: Si(OC_2_H_5_)_4_, or Si(OR)_4_ where the alkyl group R represents C_2_H_5_. Alkoxides are ideal chemical precursors for sol-gel synthesis because they react readily with water. The reaction is called hydrolysis, because a hydroxyl ion becomes attached to the silicon atom as follows:

(5) $$\text{Si}{\left(\text{OR}\right)}_{4}+{\text{H}}_{2}\text{O}\to \text{HO}-\text{Si}{\left(\text{OR}\right)}_{3}+\text{R}-\text{OH}$$

Depending on the amount of water and catalyst present, hydrolysis may proceed to completion, so that all of the OR groups are replaced by OH groups as follows:

(6) $$\text{Si}{\left(\text{OR}\right)}_{4}+4{\text{H}}_{2}\text{O}\to \text{Si}{\left(\text{OH}\right)}_{4}+4\text{R}-\text{OH}$$

Any intermediate species ((OR)_2_–Si-(OH)_2_) or ((OR)_3_–Si-(OH)) would be considered the result of partial hydrolysis. In addition, two partially hydrolyzed molecules can link together in a condensation reaction to form a siloxane [Si–O–Si] bond:

(7) $${\left(\text{OR}\right)}_{3}-\text{Si}-\text{OH}+\text{HO}-\text{Si}-{\left(\text{OR}\right)}_{3}\to [{\left(\text{OR}\right)}_{3}\text{Si}-\text{O}-\text{Si}{\left(\text{OR}\right)}_{3}]+\text{H}-\text{O}-\text{H}$$

or

(8) $${\left(\text{OR}\right)}_{3}-\text{Si}-\text{OR}+\text{HO}-\text{Si}-{\left(\text{OR}\right)}_{3}\to [{\left(\text{OR}\right)}_{3}\text{Si}-\text{O}-\text{Si}{\left(\text{OR}\right)}_{3}]+\text{R}-\text{OH}$$

Thus, polymerization is associated with the formation of a 1, 2, or 3- dimensional network of siloxane (Si–O–Si) bonds accompanied by the production of H-O-H and R-O-H species.

By definition, condensation liberates a small molecule, such as water or alcohol. This type of reaction can continue to build larger and larger silicon-containing molecules by the process of polymerization. Thus, a polymer is a macromolecule formed from hundreds or thousands of monomers. The number of bonds that a monomer can form is called its functionality. Polymerization of silicon alkoxide, for instance, can lead to complex branching of the polymer, because a fully hydrolyzed monomer Si(OH)_4_ is tetra functional; it can branch or bond in 4 different directions. Alternatively, under certain conditions (e.g., low water concentration), fewer than 4 of the OR or OH groups will be capable of condensation. Thus, relatively little branching will occur. The mechanisms of hydrolysis and condensation, and the factors that bias the structure toward linear or branched structures, are the most critical issues of sol-gel science and technology [38–44].

## 4. General Procedures Involved in the Preparation of Photocatalysts with Sol-Gel Method

The photocatalysts were prepared with the sol-gel method as described in the literatures [45,46]. In the preparation of Ti-precursor sol, titanium isopropoxide (TPIP) was dissolved in de-ionized water, and the molar ratio of H_2_O/TPIP = 51:1. After stirring vigorously for 1 min, 0.25 M HNO_3_ was added into the mixture. The weight ratio of HNO_3_:TPIP was 1.5:1. The final solution was stirred vigorously until the translucent Ti-precursor sol formed. The Ti-precursor sol was dehydrated and the alcohol was removed in a rotary vacuum evaporator (N-1000S, EYELA) in a 60 °C water bath. In the preparation of Fe-TiO_2_, the calculated amounts of the metal nitrate salts were added in the de-ionized water first, and the other processes were conducted with the same method described above. The samples were dried for 24 h at 120 °C in an oven. The appropriate amounts of samples were dissolved in water-ethanol solution (water:absolute ethanol = 3:7 in weight ratio). The weight ratio of samples:solution was 1:11 for the formation of the coating solution. Pyrex cylinder glass was used as the substrate for the thin film with a spin coating method. The coated glasses were calcined at various operating temperatures for 4 h in air flow. All chemicals used in the laboratory were purchased from Aldrich, Merck and Riedel-de Haën for titanium isopropoxide (>97%), nitrite acid (>65%), and iron nitrate nonahydrate (>99%) [47,48].

## 5. Photocatalysts

Many researchers have synthesized various photocatalysts to decompose VOCs. Table 1 shows, from the references, a summary of the various TiO_2_ photocatalysts synthesized by the sol-gel method. It is seen that most of the photocatalysts use Ti(OC_3_H_7_)_4_ as the precursor. Additionally, pure TiO_2_ and TiO_2_ with a porous material are effective under UV light; in contrast, nonmetal doped TiO_2_ photocatalysts are effective under visible light.

### 5.1. Common Photocatalysts

Common photocatalysts are semiconductors such as ZnO, GaP, TiO_2_, SiC, CdS, and Fe_2_O_3_ [3]. Among various common photocatalysts, TiO_2_ has always been the subject of work for application in environmental purification due to its low cost, non-toxicity, high oxidizing power, chemical stability, and environmentally friendly characteristics [53–56].

#### 5.1.1. Visible Light Responsive Photocatalyst

Although TiO_2_ has been widely investigated in environmental applications, it cannot effectively utilize visible light because of its large band gap of 3.2 eV. This gap corresponds to 387 nm wavelength light and high recombination between electron-hole pairs. A number of works have focused on elevating the photocatalytic activity of TiO_2_, including studies involving doping with metals [57,71], doping with nonmetals [72–76], and coupling with other supports [50,52,66–68,77–87].

Recently, the doping of TiO_2_ with nonmetals, such as C [88], N [61,62,76,89–95], S [75], P [63,74], shows significant improvement in causing photosensitization in the visible region. For these anion-doped TiO_2_ photocatalysts, these species substitute the oxygen lattice on TiO_2_ and lead to a band gap narrowing, resulting in high visible spectrum absorption. It was found that doping nonmetals into TiO_2_ can narrow the band due to the contribution of its *p* orbital [61].

P-doped TiO_2_ was prepared by a simple modified sol-gel method with hypophosphorous acid as a precursor, and it was found that P-TiO_2_ significantly increased the surface area of the photocatalyst and consequently provided a higher content of surface hydroxyl groups, thus elevating photocatalytic activity [74]. The phosphorus doping extended the spectral response’s shift into the visible region, and represented more effective photocatalytic degradation of the methylene blue (MB) and 4-chlorophenol (4CP) under visible light (>400 nm) irradiation.

Many studies have described the enhanced photocatalytic activity of TiO_2_ by nitrogen doping. There are several methods to prepare N doped TiO_2_ photocatalysts with sol-gel, such as heating TiO_2_ with NH_3_ gas flow [89], oxidatively decomposing the Ti-melamine complex [95], and using nitric acid as a source of nitrogen [62]. Light absorption shifts into the visible region was observed in N-doped TiO_2_, and the extent of light absorption in the visible region was found to depend upon the content of N in the N-doped TiO_2_ [95]. Moreover, titania was prepared through co-doping with double non-metal elements, S and N, by the sol-gel method. Thiourea was used as a nitrogen and sulfur source [75]. The S-N co-doped TiO_2_ exhibited strong absorption ability in the near UV and visible light region. The photocatalytic activities in the visible-light region are about three times higher than that of Degussa P25.

#### 5.1.2. Synthetic Composites with Metal

A lot of investigations dedicated toward improving the efficiency of photocatalysts that used solar light have been conducted. Most focus on TiO_2_-based catalysts.

It was found that metal ion doped TiO_2_ can induce visible light response. However, most of them do not show long term stability [84,96].

It was discovered that doping with transition metals, such as Fe, Pd, and Pt, in TiO_2_ could lead to the absorption of photocatalysts shifting into the visible range [71,97]. However, this treatment could also trigger considerable decreases in photocatalytic activity [71]. In addition, doping Fe^3+^ and Pb^2+^ into TiO_2_ was found to show much better benzene decomposition activity [60].

It was believed that doping with metal will enhance the trapping of electrons and inhibit electron-hole recombination during illumination. Xin *et al*. employed different doping ratios of Fe^3+^-TiO_2_ by the sol-gel method, and the results revealed that as Fe^3+^ doping content exceeded 0.03 mol%, Fe_2_O_3_ became the recombination centers of photoinduced electron-hole pairs, and the decomposition rate of rhodamine B (RhB) was reduced [98].

Colmenares *et al*. found that Pd, Pt, and Ag elevated photocatalytic activity, whereas doping with Zr and Fe enhanced activity slightly [99]. The addition of metals could be either beneficial or detrimental on such metals that decrease the electron/hole recombination rate or on the contrary behave as electron/hole recombination centers.

#### 5.1.3. Hybrid Photocatalysts

Another effective method for improving the photoactivity of TiO_2_ is via coupling with other metal oxide materials and with semiconductor that have lower energy band gaps [84]. These materials include WO_3_ [77,100], SiO_2_ [52,66,67,77,79,81,87,101], CdS [78], YFeO_3_ [68], or porous material with large surface areas (e.g., activated carbon) [50,82,83,86].

A number of studies have focused on TiO_2_/SiO_2_ photocatalysts. Guan [81] found that the TiO_2_/SiO_2_ surface has more hydrophilic activity, but less photocatalytic activity; Yu *et al*. [79] revealed that the larger the amount of SiO_2_ added, the smaller the grain size in the resultant TiO_2_/SiO_2_ composite, and the larger the surface hydroxyl content on the films. The photocatalytic activity of the TiO_2_/SiO_2_ composite thin films increases when the amount of SiO_2_ is less than 5 mol%. However, when the amount of SiO_2_ is greater than 10 mol%, the photocatalytic activity of TiO_2_/SiO_2_ begins to decrease.

The photocatalytic degradation of methylene blue was investigated by TiO_2_/PDMS (poly dimethylsiloxane) and TiO_2_/SiO_2_ films [87]. The results show that TiO_2_/SiO_2_ film exhibited higher degradation rates, which were probably due to higher crystallinity and high hydrophilicity. These qualities were further enhanced by the UV-illumination and the super hydrophilic state of the film.

TiO_2_/SiO_2_ pellets were found to have a high adsorption capacity. They can serve dual functions as a photocatalyst and as an adsorbent in the hybrid photocatalysis and adsorption system. The results also demonstrated that the porous photocatalyst with high adsorptive capacity enhanced the subsequent photocatalysis reactions and led to a positive synergistic effect. The catalyst can be self-regenerated by PCO of the adsorbed VOCs [67].

Activated carbons have been extensively studied as a support for TiO_2_ [50,82,83,86]. Li *et al*. succeeded in preparing TiO_2_-coated activated carbon with increased photocalaytic activity [82]. The carbon in activated carbon reduces TiO_2_ to form more Ti^3+^ ions, thus trapping the photogenerated electrons in the conduction band and preventing the recombination of electron-hole pairs. The photoactivity of WO_x_-TiO_2_ was significantly higher than that of pure TiO_2_, and the light absorption band tended to shift into the visible range.

Moreover, tungsten oxides doped into TiO_2_ can reduce the recombination rate of excited electrons/holes [77]. Wang *et al*. [68] reported that TiO_2_ loading on the surface of YFeO_3_ can prolong the life of electron-hole pairs and also reduce the recombination of them, thus resulting in the elevation of photocatalytic activity of TiO_2_/YFeO_3_ and the narrowing of the band gap energy.

### 5.2. Coating Methods

To increase the reaction rate of PCO in the gas-solid heterogeneous photocatalysis, it is necessary for the reaction system to provide efficient contact of excited photons with photocatalysts and gaseous reactants [52]. It was believed that fluidized beds provide better use of light and better contact between the target compound and photocatalysts [52].

In previous literature, thin film was widely employed due to its large surface area and higher photocatalytic activity (when compared with commercial TiO_2_ powder). Generally, there are two methods to prepare TiO_2_ thin films. The first one is to handle the catalysis powder by direct sintering. The other one is to form the TiO_2_ film on support by using sol-gel, chemical vapor deposition (CVD) or metal-organic CVD (MOCVD).

TiO_2_ thin films prepared by a sol-gel dip coating process were studied [87,104–106] and the dip coating had some processing parameters that reflected on the porosity and refractive index of the films [102,103]. CeO_2_-TiO_2_ was prepared by sol-gel spin-coating process [102,107], and the process was found to have more processing parameters than dip coating did, including viscosity, surface tension and amount of the sol, the speed of the substrate rotation, and the humidity and temperature of the surroundings.

### 5.3. Deactivation

It was reported that TiO_2_ might be deactivated after a period of use [52,53,68,80,108], and that the intermediates and final products produced from photocatalytic oxidation, and which cover the catalytic surface, are responsible for TiO_2_’s deactivation.

It was found that the photocatalytic activities of both TiO_2_ and AS2T2 films (T and AS indicates TiO_2_ and Al_2_O_3_-SiO_2_, respectively) decreased in several runs [109]. Arana *et al*. reported that the formation of acetates during the ethanol continuous flow degradation was the cause of the progressive deactivation of bare-TiO_2_ and Fe-TiO_2_ [110]. Cao *et al*. studied the deactivation of TiO_2_ by PCO of toluene, and it was discovered that performing the reaction at room temperature resulted in rapid deactivation of TiO_2_ catalysts due to the chemisorptions of intermediates, such as benzaldehyde and benzoic acid. The color change of the catalysts from white to yellow may be attributed to the accumulation of these carbon species covered active sites [53].

Furthermore, experiment on the photodegradation of gas phase toluene was carried out using TiO_2_/SiO_2_ [52]. The intermediate products of photodegradation of gas-phase toluene were benzaldehyde, benzoic acid, and benzyl alcohol. Benzoic acid is strongly adsorbed on the surface of the photocatalyst, and the accumulation of benzoic acid on the surface appeared to be responsible for the photocatalyst deactivation.

#### Regeneration

For real-life application, the PCO must be sustainable. It means that the photocatalysis should be reversible with respective to deactivation caused by oxidized intermediates or absorbed byproducts on the surface of these catalysts.

There are three methods used to regenerate deactivated photocatalysts: thermal regeneration [53,109,111], photocatalytical regeneration [112], and regeneration through washing [108].

Cao *et al*. regenerated the deactivated photocatalysts by raising regeneration temperature to remove intermediates from the active sites [53]. In order to completely recover the deactivated catalysts, a regeneration temperature at or above 420 °C is needed.

Sun *et al*. used two methods to regenerate the deactivated photocatalysts [111]. One was through re-calcinations at 450 °C under air flow for 3 h. The other one was through irradiation at 80 °C under the 450 W UV lamp used above with oxygen flow for 24 h.

## 6. Effect of Operational Parameters and Kinetic Models

In the PCO reaction, different operational parameters will affect the conversion of target compounds and the results of kinetic models. Table 2 shows the list of operational parameters in some references, including model compounds, VOC concentration, O_2_ concentration, operational temperature, humidity, and light source.

### 6.1. Effect of Operational Parameters

#### 6.1.1. Light Intensity

TiO_2_ is an inexpensive, stable, and non-toxic semiconductor with a large band gap and strong oxidizing power [113]. It can be activated under UV light wavelengths shorter than 388 nm due to its band gap energy of 3.2 eV [114]. The solar ultraviolet (UV) radiation is usually applied to activate the photocatalytic reaction, and the photo-oxidation on TiO_2_ surface irradiated with suitable UV radiation ideally leads to a complete mineralization of VOCs into other environmentally friendly species, such as water, carbon dioxide, and mineral acids [55,113].

It is well known that the light intensity is an essential factor in influencing the reaction rate of VOCs and the utilization ratio of energy [115]. In general, the performance of photocatalytic decomposition rate increases with rising light intensity [55]. It was found that the degradation rate of trichloroethylene (TCE) for TiO_2_-GP (TiO_2_ films coated on glass plates) increased linearly with rising light intensity [114]; however, excessive light intensity results in more electron-hole recombination. It was proposed that lower light intensity could be applied to the degradation of lower-concentration VOCs to reduce the energy losses resulting from the electron-hole recombination.

When considering the electron-hole recombination, the appropriate light intensity, *I_a_*, can be predicted by the following function [115]:

(9) $$I_{a}=mI$$

where *m* is an excess coefficient, and *I* is the light intensity.

It was worth noting that the light intensity had a great performance on the electron-hole recombination. The value of *m* is related to the influence of electron-hole formation, and it can also be observed that the electron-hole recombination increased with rising light intensity (*m* = 1). When the ratio of the used to the calculated light intensity (*m* = 1) exceeded a certain value, the electron-hole recombination would increase quickly [113,115]. Sometimes with the increase of light intensity, the increment of reaction rate would reduce, which indicated that the utilization ratio of light energy might drop.

The reactor system also influences the light intensity, and using a fluidized bed photoreactor not only brings the photocatalyst into contact with more gas, but also enhances UV-light penetration through bubbles. Kim *et al*. found that at lower gas flow rates, the effect of light intensity was negligible. In contrast, at higher gas flow rate conditions, the effect of light intensity was evident. This is because at higher gas flow rates, the bubble phase flow was increased and light transmission through the bubbles became an important factor in determining the reaction rate [116].

#### 6.1.2. Nature of the Photocatalyst

The extensive research on TiO_2_ created an expectation to use merely 3–4% UV light of the whole radiant solar energy [124]. In order to extend the applicable wavelength range, recent research has focused on doping TiO_2_. With metal or non-metal species, doping TiO_2_ is beneficial to reduce the recombination of photogenerated electrons and holes, and it can also enhance the transfer and transport of charge carriers and induce the shift of the absorption edge into the visible-light range with narrowing band gap. This can directly initiate the oxidative reaction to decompose organic pollutants [60,62,121,125,126]. Many works have indicated that photocatalyst doped by N were the most effective because its *p* states contribute to the band gap narrowed by mixing with O_2_*_p_* states [62,72,76]. Research was carried out to improve the activity of the photocatalyst by adding substance. By having a smaller band gap in comparison with TiO_2_, the substance can receive photons under visible light, form electron-hole pairs, and transport electrons to the conduction band [116,117,120].

Furthermore, some researchers have suggested that when compared with bare TiO_2_, TiO_2_ supported on adsorbent provides a higher specific surface area and is more capable of adsorbing the compounds on the effective adsorption site [50,127–131]. The enhanced decomposition rates are attributed to the increased adsorption capacity of organic substrates on the supported catalyst by adsorption site and the reduced the recombination rate of electron-hole pairs process on the surface [127,131].

#### 6.1.3. Photocatalyst Concentration

In photocatalytic oxidation, the activity of the photocatalyst not only depends upon the properties of the loading species, but also upon the amount of catalyst loaded [132]. Zhang *et al*. [122] reported that the decomposition quantities increased with the amount of metal doped and that 2.5 wt% La-doped TiO_2_ achieved the highest decomposition capacities. Such quantities then decreased with further doping. This decrease can be attributed to the fact that excessive metal will be present as an oxide on the surface of TiO_2_ under these conditions. These oxides acts as the recombination center and therefore decrease the photocatalytic ability.

The decomposition rate increases gradually with the increase of thickness. This can be attributed to the further absorption of light intensity. This means that catalyst film requires an appropriate numbers of coats to completely absorb the light. Thus, it was expected that the reaction rate would not increase with increasing coats[115]. Therefore, it is suggested that the strong impact of wavelength on the optical attenuation results in the optimal catalyst film thickness differing for different wavelengths.

#### 6.1.4. Humidity

Water vapor content plays an important role in the photocatalytic degradation of gaseous organic compounds. The presence of water in air will significantly affect the photocatalyst activity. The water vapor is a source of hydroxyl radicals on the photocatalyst surface and those surface mediated reactions deplete hydroxyl radicals if water is not sufficient for replenishment [117]. The water molecules can be transformed into hydroxyl radicals (OH•) by reacting with the photogenerated holes (H^+^) or superoxide radicals (O_2_^−^•) at the photocatalyst surface via the following reactions:

(10) $${\text{h}}^{+}+{\text{H}}_{2}\text{O}\to \text{OH}•+{\text{H}}^{+}$$

(11) $$2{\text{O}}_{2}^{-}•+2{\text{H}}_{2}\text{O}\to 2\text{OH}•+2{\text{OH}}^{-}+{\text{O}}_{2}$$

As the humidity in the reactor increases, the reaction is no longer limited by the OH• radicals, thus leading to the observed increase in the reaction rate constant [117,119].

At higher water concentrations, the competitive adsorption between water vapor and the organics on the active sites may decrease the reaction rates [53,119], indicating that water vapor can efficiently adsorb on the catalytic beads [118]. This phenomenon is called “competitive adsorption” between water vapor and contaminants on photocatalytic degradation. It was found that water vapor did not participate in the reaction, but would compete with the organics for the active sites on the TiO_2_ surface [119]. In addition, it also inhibited the reaction rate; that is, in lower humidity ranges the reaction rate only slightly decreased with increasing relative humidity, whereas it apparently decreased with the increasing relative humidity in higher range [76,120,123].

This phenomenon can be attributed to the competitive adsorption between water molecules and VOCs on the photocatalyst surface, and the blocking of humidity on the active sites of the photocatalyst surface. The increased content of moisture in the system can destroy the equilibrium, between consumption and adsorption of water vapor, which keeps stable reaction rates. Therefore, increasing adsorption of the water molecules on the surface will decrease the reaction rate of the catalyst [120,123].

Boulamanti and Philippopoulos studied 5 targ*et al*kanes from C_5_ to C_7_ under different values of relative humidity (0–90% RH). The obtained results indicate that the molecular and stereo-chemical structures of the compounds play an important role in PCO reaction [123]. It seems that the influence of water vapor in the gas-phase degradation reaction depends on the species of pollutants, as well as on the concentrations of both VOCs and humidity [123].

#### 6.1.5. Reaction temperature

The effect of temperature on photocatalytic activity impacts not only the kinetic reaction, but also the adsorption of contaminants [117,133,134]. To determine the reaction rate, the temperature dependence of the kinetic parameter *k*_obs_, and the Arrhenius equation can be expressed as follows [133]:

(12) $$k_{\text{obs}}=A\;\text{exp}(-\frac{Ea}{RT})$$

where *k*_obs_ is the kinetic parameter (min^−1^), *A* is the frequency factor (min^−1^), *E*a is the activation energy (kcal mol^−1^), *T* is the temperature (in Kelvin), and *R* is the gas constant (1.987 × 10^−3^ kcal mol^−1^ K^−1^).

The degradation efficiency usually increased with increasing temperature [133,134]. In previous research, the reaction rate was increased at 35–70 °C, instead of 70–100 °C, and it may be ascribed to the shorter contact time and weaker interaction between the aromatic gas molecules and the adsorption site on the surface. This leads to the limitation of the adsorption tendency under the mass transfer process [117].

At low metal concentration, the adsorption of cerium dopant was affected more significantly by temperature in comparison with that of lanthanum. Moreover, excess lanthanide could inhibit the benzene adsorbption ability [134]. It was concluded that the degradation rate of the photocatalyst was dependent upon the reaction temperature and catalyst species [117,134], and that the activation energy varied slightly with different flow rates [133].

#### 6.1.6. Oxygen

In the absence of oxidizing and reducing agents, the hole-electron will reach a balanced condition. It is well known that oxygen is an effective conduction band electron acceptor [120]. Molecular oxygen pre-adsorbed onto the surface of the photocatalyst can instantly trap the interfacial electron of the photocatalyst so as to suppress the hole-electron recombination by the presence of residual oxygen in the reaction system [118,120]. The decomposition rate increases with increasing oxygen concentration. The competitive adsorption between pollutants and molecular oxygen is not strong, but it should be noticed that the role of adsorbed oxygen molecules is not limited to the electron-trapping.

Zhang *et al*. [66] investigated the photodegradation of four carbonyl compounds. The experimental results show that the photocatalytic degradation of four carbonyl compounds was ineffective in the absence of oxygen, and that the photodegradation gradually increased with increasing oxygen concentration. But there was no further increase of the photocatalytic degradation efficiencies when oxygen concentration was greater than 30%.

#### 6.1.7. Poison Effect

When different pollutants were mixed with the pollutants of photo-degradation, they could compete with other pollutants or increase the decomposition rate. Consequently, usually, the PCO reaction rate should be inhibited [135,136].

The photodegradation rate of pollutants can be influenced by the presence of NO, SO_2_, and VOCs [135]. The effect of NO on formaldehyde conversion is due to the hydroxyl radicals generated from the photodegradation of NO. The impact of the presence of SO_2_ on the photodegradation of formaldehyde sulfate ion is to compete with formaldehyde for adsorption sites on the TiO_2_ surface.

### 6.2. Kinetic Models of Photocatalytic Oxidation Reaction Process

The reaction rate is dependent on the experimental conditions (experimental setup design, irradiation conditions, and inlet concentration) and also on the competitive mechanisms of photochemical reactions, and adsorption [137].

The Langmuir-Hinshelwood (L-H) model has been widely used for VOCs catalytic reaction rate equations in gas-phase and liquid-phase photocatalysis for a surface-catalyzed reaction [55,67,80,124,127,138,139,140]. When the concentrations of water and oxygen remain constant, the equation model can be presented as follows to determine the reaction rate:

(13) $$r=-\frac{dC}{dt}=\frac{kK_{LH}C}{1+K_{LH}C}$$

where *r* is the reaction rate (mg m^−3^ min^−1^), *C* is the initial concentration (mg m^−3^), *k* is the reaction rate constant (mg m^−3^ min^−1^), and *K*_LH_ the Langmuir adsorption constant (m^3^ min^−1^) related to the limiting rate of reaction at maximum coverage for the experimental conditions. Table 3 shows the comparison of Langmuir adsorption constants and reaction rate constants of reference [80,123,139,140].

Boulamanti and Philippopoulos [123] studied the photocatalytic degradation of target gas. Considering the influence of the water vapor, the equation can be written as follows:

(14) $$r=k\frac{K_{\text{LH}}C}{1+K_{\text{LH}}C+K_{w}C_{w}}$$

where *K*_w_ is the Langmuir adsorption constant reflecting the proportion of water vapor molecules, which cling to the catalyst surface. *C*_w_ (mg m^−3^) is the gas-phase concentration of the water vapor.

The photocatalytic degradation of target gas can be described as the reaction on the surface of catalyst as follows:

(15) VOC+S↔VOC*S

(16) W+S↔W*S

(17) $$\text{VOC}*\text{S}+{\text{aO}}_{2}\to {\text{bCO}}_{2}(\text{g})+\text{cW}(\text{g})+\text{S}$$

where S is a vacant active site, while VOC*S and W*S represent the absorbed species on the catalyst surface. The complete phenomenon is assumed to be a rate-limiting reaction.

Zhang and Liu investigated the kinetics of photocatalytic degradation of VOCs on TiO_2_ with and without ozone [80]. The results indicate that the ozone could compete with hexane to scavenge hydroxyl radicals on the photocatalyst surface. In both the TiO_2_/UV and O_3_/TiO_2_/UV processes, the decomposition of hexane had a good agreement with the L-H model; experimental results of O_3_/UV did not adapt the L-H model since it was not a surface reaction.

Vincent *et al*. used two different L-H models; one is the “*simple LH model*” (Equation 13), and the other is the “*two-site model*” (Equation 14) [140]. The 1-propanol conversion was found to be partially limited by the adsorption of intermediates on the catalyst surface and the profile of 1-propanol did not strictly follow the rate form of the “*simple LH model*”. The adsorbed intermediates could block the reactive sites at the catalyst surface and inhibit the photocatalytic degradation of 1-propanol. The “*simple LH model*” was inadequate to fit the 1-propanol conversion curve and the best fit could only be obtained if the intermediates were included. It was noticed that the 1-propanol conversion decreased slightly with increasing illumination time. This trend could be attributed to the reversible deactivation of the photocatalyst, which is well known in this type of reaction (Equation 13) or to a photostationary state establishment. For the reasons mentioned above, the photocatalyst was regenerated after each experiment in order to recover its initial activity.

## 7. Summary

The sol-gel method was introduced in this study along with a summary of its inherent advantages. The fundamental chemical reactions in the sol-gel process were performed. The mechanisms of hydrolysis and condensation, and the factors that bias the structure toward linear or branched structures are the most critical issues of sol-gel science and technology. The general procedures including the preparation of photocatalysts with the sol-gel method were also investigated in the study.

A review of PCO purification of indoor VOCs was carried out. Although various photocatalysts with high photocatalytic activity and visible light response were prepared, relatively few investigative efforts have been made toward practical applications (*i.e.*, indoor air cleaner or products), which may be due to the instability of these photocatalysts. Among the various photocatalysts, TiO_2_ is the most commonly used material in the PCO of various VOCs.

The effects of operational parameters and kinetic models associated with PCO reactions have been discussed. Some experimental evidence indicates that the operational parameters change with different reaction conditions (*i.e.*, light intensity, nature of the photocatalyst, photocatalyst concentration, humidity, pollutant concentration, temperature, oxygen concentration and poison effect). The kinetic model that ignores the influence of completed adsorption among all the compounds and the water vapor concentration would only be correct for the individual experiment. Future studies assessing kinetic parameters for photocatalysts should make a specific effort to build up the criteria through which to evaluate their photocatalytic performance. This can be done by conforming the reaction conditions and the selected kinetic models to be consistent.

## Figures and Tables

**Table 1:** Summary of various photocatalysts.

**Catalyst**	**Precursor**	**Mole ratio**	**Model compounds**	**Light source**	**Ref.**
Fe-TiO_2_	Ti(OC_3_H_7_)_4_	0.05, 0.5, 5	Dichloromethane	UV lamp (λ_max_ = 365 nm)	[48]
N-TiO_2_	Ti(OC_3_H_7_)_4_	-	Toluene, Isopropanol	UV lamp (λ = 364.2 nm)	[49]
				Visible light (λ_max_ = 611 nm)	
TiO_2_	Ti(OC_4_H_9_)_4_	-	Phenol	UV lamp (λ_max_ = 365 nm)	[50]
Cr-TiO_2_/AC	Ti(OC_4_H_9_)_4_	0, 0.2, 0.4, 0.6wt%, active carbon 40 g	EDTA	UV lamp (λ = 320∼400 nm)	[51]
TiO_2_/SiO_2_	Ti(OC_3_H_7_)_4_	-	Toluene, Xylene	UV lamp (λ = 315∼400 nm)	[52]
TiO_2,_ Pt-TiO_2_	Ti(OC_3_H_7_)_4_	0.50%	Toluene	UV lamp	[53]
TiO_2_	Ti(OC_3_H_7_)_4_	-	Trichloroethylene	Fluorescent light	[54]
TiO_2_	Ti(OC_3_H_7_)_4_	-	Formaldehyde	UVA lamp (λ = 365 nm)	[55]
TiO_2_, TiO_2_/SiO_2_	Ti(OC_3_H_7_)_4_	1, 4, 9	Formaldehyde	UV-A lamp	[56]
N-TiO2,	Ti(OC_3_H_7_)_4_	-	Methanol, Ethanol	UV lamp (λ_main_ = 350 nm)	[57]
Pt-TiO_2_	Ti(OC_3_H_7_)_4_	-	Benzene, Ethanol	UV lamp, Fluorescent lamp	[58]
Fe(III)-doped TiO_2_	Ti(OC_3_H_7_)_4_	-	Ethanol	Fluorescent light	[59]
Fe^3+^-TiO_2_, Pb^2+^-TiO_2_	Ti(OC_4_H_9_)_4_	-	Trichloroethylene, Chloroform, Dichloromethane, Toluene, Benzene, Carbon Tetrachloride	UV light (λ_main_ = 253.7 nm)	[60]
N-TiO_2_	-	-	Acetaldehyde	Fluorescent light	[61]
N-TiO_2_	C_12_H_28_O_4_Ti	-	Trichloroethylene	Visible light (λ = 420 nm)	[62]
P-TiO_2_	Ti(OC_3_H_7_)_4_	0.01, 0.05, 0.1, 0.2 and 0.3	Ethanol	UV lamp (λ_max_ = 254 nm)	[63]
N-Ni /TiO_2_	Ti(OC_3_H_7_)_4_	atomic ratios: N(0.010)TiO_2_, Ni(0.015)TiO_2_, N(0.010)Ni(0.015)TiO_2_	Formaldehyde	Visible light (λ > 400 nm)	[64]
V-modified, N-TiO_2_, TiO_2_	Ti(OC_3_H_7_)_4_	-	Acetic acid	Xenon Lamp (λ = 365 nm)	[65]
TiO_2_/SiO_2_	Ti(OC_3_H_7_)_4_	-	Propionaldehyde, Acetone, Acetaldehyde, Formaldehyde	UV lamp (λ_max_= 365 nm)	[66]
TiO_2_/SiO_2_	Ti(OC_4_H_9_)_4_	-	Toluene	UV lamp (λ_max_ = 365 nm)	[67]
TiO_2_/YFeO_3_	Ti(OC_4_H_9_)_4_	2 w%	Benzene	UV lamp (λ_max_ = 365 nm)	[68]
TiO_2_/Al_2_O_3_–SiO_2_	Ti(OC_4_H_9_)_4_	Al_2_O_3_ : SiO_2_ = 3:2	Acetaldehyde	UV lamp (λ_main_ = 253.7 nm)	[69]
N–SiO_2_/TiO_2_	Ti(OC_3_H_7_)_4_	SiO_2_/TiO_2_ = 0.05, 0.10, 0.15, 0.2, 0.3	Ethylene	Visible light (λ > 420 nm)	[70]

**Table 2:** Experimental parameters of the influence factors in the PCO reaction process.

**Photocatalyst**	**Model compounds**	**Influence factor of reaction rate**					**Ref.**
		**VOCs conc.**	**O_2_ conc.**	**Temp.**	**Humidity**	**Light source**	
**N-doped TiO_2_ and TiO_2_**	Ethyl benzene, o,m,p-Xylenes, Toluene	100 ppb	-	19–25 °C	10–90%	fluorescent daylight lamp	[76]
**ZrO_2_-TiO_2_**	Propane, Isobutene, n-Butane	∼1,000 ppmv	-	35–100 °C	2–60%	UV light	[117]
**TiO_2_**	1-Butanol, 1-Butylamine	900–5,000 mg m^−3^	30%	30 °C	saturated	xenon-chloride (XeCl) excimer lamp, medium pressure mercury lamp	[118]
**Degussa P-25 TiO_2_**	Benzene	250–450 ppmv	-	100–200 °C	13,500–27,500 ppmv	UV light	[119]
**TiO_2_/SiO_2_**	Formaldehyde, Acetaldehyde, Propionaldehyde, Acetone	20 μmol m^−3^	0–100%	-	4–80%	UV light	[120]
**Al/TiO_2_,TiO_2_**	Benzene	100 ppm	300 mL min^−1^	40 °C	0–10wt.%	UV-light	[121]
**La-doped TiO_2_**	Benzene	200 ppm	-	25 °C	25%	UV-light	[122]
**TiO_2_**	Pentane, i-Pentane, Hexane, i-Hexane, Heptane.	Pentane (90.2 ppm), i-Pentane (24 ppm), Hexane (107.5 ppm), i-Hexane (78.8 ppm) Heptane (104.8 ppm)	24%	-	0–90%	Hg lamp	[123]

**Table 3:** The comparison of the kinetics data from the literature.

**Photocatalyst**	**Pollutants**	***k*(1)**	***K*_LH_(2)**	**Ref.**
**TiO_2_**	Hexane	86.2 (mg m^−3^ min^−1^)	0.0508 (m^3^ mg^−1^)	[80]
	Pentane	1.81×10^−7^ (mol m^−2^ s^−1^)	1.14×10^−4^ (m^3^ mol^−1^)	
	i-Pentane	1.97×10^−7^ (mol m^−2^ s^−1^)	1.51×10^−4^ (m^3^ mol^−1^)	
**TiO_2_**	Hexane	2.16×10^−7^ (mol m^−2^ s^−1^)	1.25×10^−4^ (m^3^ mol^−1^)	[123]
	i-Hexane	2.48×10^−7^ (mol m^−2^ s^−1^)	1.54×10^−4^ (m^3^ mol^−1^)	
	Heptane	3.03×10^−7^ (mol m^−2^ s^−1^)	2.83×10^−4^ (m^3^ mol^−1^)	
**TiO_2_**	Formaldehyde	46.72 (mg m^−3^ min^−1^)	0.0268 (m^3^ mg^−1^)	[139]
**TiO_2_**	1-Propanol	1024 (ppm min^−1^)	0.014 (ppm^−1^)	[140]

(1): Rate constant

(2): VOCs adsorption constant
